# Evaluating differential developmental trajectories to adolescent-onset mood and psychotic disorders

**DOI:** 10.1186/1471-244X-13-303

**Published:** 2013-11-12

**Authors:** Ian B Hickie, Daniel F Hermens, Sharon L Naismith, Adam J Guastella, Nick Glozier, Jan Scott, Elizabeth M Scott

**Affiliations:** 1 Clinical Research Unit, Brain and Mind Research Institute, University of Sydney, 100 Mallett Street, Camperdown, NSW 2050, Australia; 2 Academic Psychiatry, Institute of Neuroscience, Newcastle University, Newcastle, UK; 3 Centre for Affective Disorders, Institute of Psychiatry, London, UK; 4 Academic Psychiatry, Wolfson Unit, Centre for Ageing & Vitality, Newcastle , UK; 5 School of Medicine, University of Notre Dame, Sydney, Australia

**Keywords:** Youth, Neuropsychology, Clinical staging, Sub-syndromal, Phenotype, Illness trajectory

## Abstract

**Background:**

It is an open question as to whether differential developmental trajectories, potentially representing underlying pathophysiological processes, can form the basis of a more useful typology in young persons who present for mental health care.

**Methods:**

A cohort of 605 young people was recruited from youth mental health services that target the early phases of anxiety, mood or psychotic disorders. Participants were assigned to one of three clinical sub-types (anxious-depression; mania-fatigue; developmental-psychotic) according to putative developmental trajectories.

**Results:**

The distribution of subtypes was: 51% anxiety-depression, 25% mania-fatigue and 24% developmental-psychotic, with key differences in demographic, clinical, family history and neuropsychological characteristics. When analyses were limited to 286 cases with ‘attenuated’ or sub-threshold syndromes, the pattern of differences was similar. Multinomial logistic regression demonstrated that compared to the developmental-psychotic subtype, both the mania-fatigue and anxiety-depression subtypes were younger and more depressed at presentation, but less functionally impaired. Other discriminating variables between the developmental-psychotic and mania-fatigue sub-types were that the latter were significantly more likely to have a family history of bipolar disorder but have less likelihood of impaired verbal learning; whilst the anxious-depression group were more anxious, more likely to have a family history of depression, and had a higher premorbid IQ level.

**Conclusions:**

This cross-sectional evaluation provides preliminary support for differing developmental trajectories in young persons presenting for mental health care. Prospective follow-up is needed to examine the predictive validity of this approach and its relationships to underlying pathophysiological mechanisms.

## Background

The optimal meta-structure for differentiation of the major psychotic, bipolar and unipolar mood disorders for use in either clinical practice or clinical or related genetic, neurobiological or psychological research remains controversial [1-13]. The failure to resolve these issues may have contributed significantly to the lack of progress in recent years in developing new treatments or objective markers of illness risk, progression or response to treatment [8-13]. Progress in this area may well rely on more novel cross-sectional, longitudinal or intervention studies that are not simply limited to cohorts that are restricted to subjects who meet existing current criteria for caseness or arbitrary diagnostic algorithms that not only demonstrate low reliability [14] but poor validity [13,15]. While such studies of broad populations are uncommon in the specialist mental health literature, they are of considerable relevance not only for discovery purposes but also to those who provide ongoing primary or general health care to these more representative but less-diagnostically differentiated patient cohorts [8,9,13,16].

A further consideration is whether such novel studies are better conducted closer to onset of the major disorders, where confounding factors arising as a result of chronicity, prolonged exposure to treatments or developments of secondary morbidities are less important [15,17-21]. When assessed early in the illness course many symptom clusters do not yet meet the diagnostic thresholds employed in current classification systems [16]. As such, the clinical phenotypes of the early stages of major mood or psychotic disorders are not easily distinguished and attempting to classify cases according to conventional diagnostic criteria and/or predict the illness trajectory may be particularly unreliable [16,22]. In that context, our own recent studies have increasingly focused on adolescents and young adults who present in the early phases of major mental disorders with admixtures of anxiety, depressive, hypomanic, psychotic or substance misuse-related symptoms [13,18].

Researchers have also increasingly highlighted the added value of incorporating clinical staging alongside traditional diagnostic formulations, especially for assessing those initial, less specific clinical presentations that are common in young people when they first present for specialist care [15,23-27]. We have established a clinical staging model that classifies cases according to disease progression from an asymptomatic, at risk stage (stage 0), to non-specific clinical presentations (stage 1a) through to more specific, sub-threshold presentations (stage 1b), and syndromes meeting diagnostic criteria (stage 2, 3, or 4) [16,28,29]. Although our model is independent of traditional diagnoses there are several variations of staging models for mental disorders in use currently (see Cosci and Fava [30]). Other approaches propose separate models for each major set of disorders (including schizophrenia, unipolar depression, bipolar and alcohol use disorders), despite the high rate of current and lifetime comorbidity between these conditions. Whilst the clinical staging approach is gaining acceptance, it may now be apposite also to explore the utility of employing clinical sub-groupings that more closely reflect differential developmental, and possibly underlying pathophysiological, pathways [13].

We have proposed that there are at least three pathways are common and observable in youth mental health cohorts [13], and that each can be linked to particular clinical phenotypes in young people. The proposed pathways, which may reflect underlying pathophysiological mechanisms, emphasize neuro-developmental impairments, circadian dysregulation or heightened sensitivity (i.e. stress-reactivity) in the ‘fear’ circuitry. We have assigned the following labels to each clinical phenotype, namely: developmental-psychotic (DEV-PSY), mania-fatigue (MAN-FAT) and anxiety-depression (ANX-DEP) sub-types.

Importantly, the DEV-PSY group is at the heart of current research domain criteria (RDoC) initiative proposed by the NIMH, and is hypothesized to be associated with autism, schizophrenia and/or psychotic phenomena [31-34]. It is also consistent with other meta-structures that were proposed for redevelopment of international diagnostic systems [1,2]. As we hypothesize a different illness trajectory for the DEV-PSY and MAN-FAT subgroups, it is important to note that any cases with manic like symptoms (manic, hypomanic or brief hypomanic phenomena) are preferentially allocated to the MAN-FAT sub-type irrespective of past or current evidence of psychotic or other phenomena. Threshold and sub-threshold depressive syndromes are sub-divided using evidence from ‘probabilistic’ and dimensional models that differentiate between presentations that are more likely to follow a bipolar (MAN-FAT) as compared to a unipolar (ANX-DEP) course [35,36]. We employ the fatigue (FAT) label as the individual characteristics that most frequently differentiate the trajectories are the atypical features of depression, especially reduced activation and energy, and increased need for sleep, etc. [37-39]. The revised DSM-V criteria recognize the importance of activation as a cardinal feature of bipolarity, which is often a manifestation of underlying circadian rhythm dysregulation [40,41].

The final (and here residual) sub-type comprises individuals who frequently reported childhood anxiety, but later show evidence of heightened stress-sensitivity and an evolving depressive disorder (but without evidence of a ‘fatigue’ profile); this is the ANX-DEP sub-type [13]. This most closely reflects the traditional emphasis in unipolar mood disorders on genetic factors that underpin stress-sensitivity and early onset anxiety and depressive disorders [42-46]. Additionally, it encapsulates models of the neural circuitry of fear responses or glucocorticoid-dependent arousal and aberrant or prolonged stress responses [45,47-51]. From a therapeutic perspective, it emphasizes those depressive disorders that are best managed by reduction of life-long and predisposing anxiety – by relevant psychological (e.g. CBT) or pharmacological (e.g. SSRIs) interventions.

The first step in testing the validity of these proposed clinical sub-types is to examine:

(i) Which demographic, clinical, family history, childhood phenotype, and neuropsychological characteristics are most strongly associated with cases allocated to each sub-type;

(ii) If the same patterns of clinical and neuropsychological characteristics are evident in cases presenting with a sub-threshold or attenuated clinical syndrome (i.e. stage 1b); and,

(iii) Which combination of variables best distinguish between the MAN-FAT, DEV-PSY and ANX-DEP sub-types after controlling for symptoms and functional impairment at the time of presentation to the youth mental health services.

## Methods

### Participants

With approval from the University of Sydney Human Research Ethics Committee, consecutive cases presenting with mood and/or psychotic symptoms to youth mental health services in and around Sydney were invited to participate in a detailed multi-level clinical assessment. Individuals were included in the study cohort if they: (i) were willing and able to give written informed consent (or parental consent was obtained e.g. for those aged <16 years); (ii) presented to the clinical services with sub-threshold mood and/or psychotic symptoms or with a mood or psychotic syndrome; *and* (iii) agreed to participate in longitudinal follow-up interviews (including further clinical, sleep, neuropsychological and neuro-imaging studies).

Exclusion criteria for all potential participants were: medical instability or lack of capacity to give informed consent (as determined by a psychiatrist), history of neurological disease (e.g. tumor, head trauma, epilepsy), medical illness known to impact cognitive and brain function (e.g. cancer, ECT in last 3 months), and/or clinically evident intellectual and/or developmental disability, and/or insufficient English to participate in the research protocol.

All patients were receiving clinician-based case management and relevant psychosocial interventions at the time of assessment. Additionally, patients who were treated with psychotropic medications were assessed under ‘treatment as usual’ conditions, whereby their normal medications were not altered. At the time of assessment, 27% (163/605) of patients were not taking psychotropic medications; 44% (267/605) were taking an anti-depressant; 38% (229/605) an antipsychotic medication; and 13% (80/605) were taking a mood stabiliser.

### Allocation to clinical sub-types

An independent reviewer (DH) allocated cases to one of the three proposed sub-groups on the basis of the description of the clinical presentation alone (i.e. without reference to any information from the structured research assessment). Any cases presenting with depressive symptoms only, with no evidence of psychotic or manic features, and no indication of atypical symptoms were allocated to the ANX-DEP group. The reviewer was blinded to all other clinical and historical information, so cases were excluded (N = 86) from this cross-sectional study if they could not be allocated to one of the three sub-types.

### Assessment procedure

Structured clinical interviews were undertaken by trained researchers (psychiatrists, psychologists and neuropsychologists) to establish the following:

a) *Demographics and key characteristics*: age, gender, and years of education were recorded. In addition, age at onset of psychiatric symptoms was assessed. Egeland et al. [52] noted that there are several definitions that can be used as a proxy measure for age at onset, but as the assessment is usually retrospective, all estimates represent approximations and the definition that is chosen will represent a compromise between sensitivity and reliability (e.g. personal reports will usually report earlier age at onset than case records, but the former may be subject to recall bias and the latter may under-estimate delays in help seeking). For the purposes of this study we report age at onset according to the age at first ever clinical presentation for treatment of a mental health problem (which is amenable to independent confirmation).

b) *Personal and family history of mental disorders*: the primary clinical diagnosis was assessed using DSM-IV criteria, and lifetime history of other axis 1 disorders such as Autistic Spectrum and Attention Deficit Hyperactivity Disorder (ADHD) were recorded. Current comorbidities, such as substance misuse disorder, were also assessed using general questions supplemented by the 10-item Alcohol Use Disorders Identification Test (AUDIT; [53,54]). We noted age at first exposure to, and self-reported use of, nicotine, cannabis and/or alcohol. Family history of five mental disorders (anxiety; depression; bipolar; schizophrenia/schizophreniform; substance/alcohol misuse) and of suicide was recorded and where possible independent corroboration was obtained. The assessment information was also used to determine the clinical stage of illness (see [13] and [16]). In a small proportion of cases (5%), clinical stage could not be accurately assessed, so no rating was assigned. In this study, cases rated as stage 1b were identified separately to allow comparison of the characteristics of attenuated and sub-threshold versus threshold syndromes (stage 2 and above).

c) *Ratings of current symptoms and level of distress*: The interviewer completed the 24-item Brief Psychiatric Rating Scale (BPRS-24) [55] to quantify general psychiatric symptoms (this assessment was undertaken as near as possible to the neuropsychological testing). Manic symptoms were assessed using the Young Mania Rating Scale (YMRS; [56]), whilst depressive symptoms were assessed using the 17-item Hamilton Depression Rating Scale (HDRS-17) [57], In addition, suicidal ideation is reported separately as a dichotomy (yes/no). Psychological distress and fears about general social interactions (and consequent risk of social avoidance) were assessed using two established self-ratings: the Kessler-10 (K-10) [58] and the Social Interaction Anxiety Scale (SIAS) [59].

d) *Social functioning*: the clinician completed the observer-rated social and occupational functioning assessment scale (SOFAS) [60]; this is rated from 0 to 100 with lower scores indicating greater impairment.

e) *Neuropsychological profile*: premorbid intelligence (‘predicted IQ’) was estimated on the basis of performance on the Word Reading subtest of the Wide Range Achievement Test 4 [61] for those 12 to 15 years old or the Wechsler Test of Adult Reading [62] for those aged 16 to 30 years old. A sub-set of neuropsychological tests were then selected on the basis of their previously established sensitivity to subtle cognitive changes in young people with mental disorders [63]. These were: ‘psychomotor speed’, assessed using the Trail-Making Test, part A (TMT A); ‘mental flexibility’, assessed by part B (TMT B) [63,64]; ‘verbal learning and memory’, assessed by the Rey Auditory Verbal Learning Test (RAVLT) [64] using the sum of trials RAVLT A1-A5 (learning), and 20-minute delayed recall by RAVLT A7 (memory).

### Statistical analyses

Statistical analyses were performed using SPSS for Windows 20.0. Cases with missing data were excluded list-wise from the analyses. All statistical tests were 2-tailed and used a significance level of *α* = .01. Group differences in demographic, clinical and neuropsychological variables were assessed via ANOVA or chi-square tests where relevant. Analyses of differences between sub-types were repeated for a selected sample of cases rated as stage 1b or stage 2+ (stages 2, 3, or 4). If equality of variance was compromised (according to Levene’s test) the corrected degrees of freedom and p-values were reported. To control for the effects of age, neuropsychological variables were converted to ‘demographically corrected’ standardized scores (i.e. z-scores) using established norms for TMT [64] and RAVLT [65]. Prior to analyses, outliers beyond ± 3.0 z-scores for each neuropsychological variable were curtailed to values of +3.0 or −3.0 (depending on the direction) so that the between-group tests were not skewed by extreme scores [63]. (Outliers across the four variables comprised <10% of the sample.)

Multi-nomial logistic regression (MNLR) was used to assess the combination of factors and variables that best differentiated between clinical subtypes. The DEV-PSY group was selected as the reference category and the forward (likelihood ratio) procedure was used for the regression. Variables were entered into the model if they had p values lower than 0.01.The variables included in the model were: gender, age at onset, age at presentation to youth services, predicted IQ, and ratings on the HDRS, K-10, SIAS, and SOFAS. Neuropsychological measures (TMT-A; TMT-B; RAVLT sum score, RAVLT A7) and family history of depression, anxiety, bipolar and schizophrenic disorders all met inclusion criteria. Predictors of sub-group membership are reported using B, exp(B), 95% confidence intervals (95% ci) and significance levels (p values). The Cox and Snell pseudo R^2^ statistic is reported as an indicator of the amount of variance explained by the regression model.

## Results

The study sample comprised of 605 individuals with a mean age of 19.9 (± 4.3; 12 to 30 years); about 50% (n = 304) were female. Just over half of the study participants were classified in the ANX-DEP (51%, n = 310) group, with about a quarter in either the MAN-FAT (25%; n = 152) or DEV-PSY (24%; n = 143) groups. While these characteristics closely reflect the demographics and case mix of the clinical services from which the cohort was recruited, study participants demonstrated slightly higher symptom levels [66].

As shown in Table 1, the DEV-PSY sub-group is predominantly male and is older at presentation to youth mental health services and at first ever contact with clinical services than the other groups. Although the overall level of psychiatric symptoms (as measured on the BPRS) is similar to other groups, the DEV-PSY group had less severe clinician-rated depressive symptoms (HDRS), lower levels of suicidal ideation and lower self-rated levels of social anxiety (SIAS) or distress (K-10). There were no between group differences in education level, but the DEV-PSY group had a significantly lower premorbid IQ (reflected by predicted IQ) and a lower level of clinician-rated social and occupational functioning (SOFAS).

**Table 1:** Demographic and clinical profile of cases classified as: (i) ‘anxious-depression’ (ANX-DEP); (ii) ‘mania-fatigue’ (MAN-FAT); or (iii) ‘developmental-psychotic’ (DEV-PSY)

	***ANX-DEP (n* = *310)***	***MAN-FAT (n* = *152)***	***DEV-PSY (n* = *143)***	***Significance test***
				**χ2 or ANOVA *(2, df) [p]***
Female, % (n)	53.0% (n = 165)	66.4% (n = 101)	25.9% (n = 37)	χ2 (605) = 50.2 **[.000]**
Age in yrs (Mean ± SD)	19.2 ± 4.3	20.2 ± 4.0	21.1 ± 4.6	F (604) = 10.7 **[.000]**
Age at 1^st^ treatment, in yrs	14.5 ± 4.3	14.4 ± 3.5	17.5 ± 5.4	F (251.8) = 15.8 **[.000]**
Education, in yrs	11.4 ± 2.8	11.9 ± 2.4	11.5 ± 2.6	F (549) = 2.2 [.112]
Predicted IQ	103.0 ± 10.3	101.4 ± 10.2	97.1 ± 12.1	F (500) = 12.1 **[.000]**
BPRS total	39.7 ± 10.0	41.0 ± 10.1	39.4 ± 10.6	F (536) = 1.1 [.333]
HDRS total	12.4 ± 6.8	12.1 ± 6.9	8.8 ± 5.9	F (275.9) = 14.7 **[.000]**
YMRS	3.1 ± 4.4	6.8 ± 8.6	2.4 ± 3.7	χ2 (264) = 10.4^#^**[0.005]**
Suicidal ideation	38.7% (105/271)	35.2% (50/142)	20.2% (26/129)	χ2 (542) = 13.9 **[.001]**
Distress (K-10 total)	27.3 ± 7.7	27.2 ± 8.4	22.8 ± 8.7	F (486) = 12.7 **[.000]**
Social anxiety (SIAS total)	39.0 ± 16.6	33.8 ± 18.4	31.8 ± 15.9	F (436) = 7.8 **[.000]**
SOFAS	60.4 ± 11.6	61.4 ± 11.5	56.3 ± 12.3	F (553) = 7.6 **[.001]**

**Note**: SOFAS = Social and Occupational Functioning Assessment Scale; HDRS = Hamilton Depression Rating Scale; BPRS = Brief Psychiatric Rating Scale; K-10 = Kessler-10; SIAS = Social Interaction Anxiety Scale; YMRS = Young Mania Rating Scale; ^#^Kruksal Wallis test.

As shown in Table 2, neuropsychological testing demonstrated significant group differences in processing speed, mental flexibility, and verbal learning and memory tasks. For each assessment, the DEV-PSY group performed poorly as compared to the ANX-DEP and MAN-FAT groups (who were very similar to each other). Deficits in the DEV-PSY group were particularly marked for tests of mental flexibility and verbal learning and memory (z-score < −1.0).

**Table 2:** Neuropsychological profile of cases classified as: (i) ‘anxious-depression’ (ANX-DEP); (ii) ‘mania-fatigue’ (MAN-FAT); or (iii) ‘developmental-psychotic’ (DEV-PSY)

**Mean z-scores (± standard deviation)**	***ANX-DEP (n* = *310)***	***MAN-FAT (n* = *152)***	***DEV-PSY (n* = *143)***	***Significance test***
				***ANOVA (2, df) [p]***
Psychomotor speed (TMT-A)	0.0 ± 1.0	0.1 ± 1.0	−0.4 ± 1.0	F (517) = 7.1 **[.001]**
Mental flexibility (TMT-B)	−0.5 ± 1.3	−0.4 ± 1.3	−0.8 ± 1.3	F (512) = 3.6 [.029]
Verbal learning (RAVLT sum)	−0.2 ± 1.3	−0.1 ± 1.2	−1.1 ± 1.3	F (514) = 26.2 **[.000]**
Verbal memory (RAVLT A7)	−0.1 ± 1.2	−0.3 ± 1.3	−1.0 ± 1.3	F (513) = 23.9 **[.000]**

About half of study participants (286 of the 575 with a clinical stage assigned) were rated as being at stage 1b at the time of assessment; a significantly higher proportion of cases in the ANX-DEP (n = 174; 56%) and MAN-FAT (n = 82; 53%) groups were regarded as presenting with sub-threshold syndromes compared to the DEV-PSY group (n = 28; 20%) (X^2^ = 56.2; df = 2; p < 0.001). Comparison of clinical and neuropsychological profiles between cases rated as stage 1b compared to stage 2+ suggested that between group differences were evident for stage 1b cases with regards to age at presentation, age at first treatment, years in education and predicted IQ; impairments in functioning and general behavioral disturbance were less marked in the stage 1b compared to stage 2, but the pattern of differences was consistent. There were marginal changes in gender distribution (Female: ANX-DEP = 58%; MAN-FAT = 72%; DEV-PSY = 29%), depression severity (mean HDRS: ANX-DEP = 12.9; MAN-FAT = 12.6; DEV-PSY = 9.6), and distress levels (mean K-10: ANX-DEP = 27.9; MAN-FAT = 28.5; DEV-PSY = 23.9). The pattern of significant neuropsychological differences remained the same, although the overall levels of impairments were less marked in the stage 1b compared to stage 2+ in the DEV-PSY group (data available from the corresponding author).

As shown in Table 3, there was a significant difference (p < .05) among the tripartite sub groups in the proportion of patients who were not taking any psychotropic medications at the time of assessment with the ANX-DEP group having the highest proportion (i.e. 31%). Chi-square tests also revealed highly significant differences among the subgroups in the proportions of patients who were taking any anti-depressant, any anti-psychotic and any mood stabiliser (see Table 3). Approximately half of the ANX-DEP and MAN-FAT groups were taking any anti-depressant medication compared to only one-quarter of the DEV-PSY group. In terms of any anti-psychotic use, the MAN-FAT and DEV-PSY groups were two to three times more likely to be taking this medication compared with those in the ANX-DEP group (at 22%). While less than 10% of the both the ANX-DEP and DEV-PSY groups were currently taking any mood stabiliser, more than 30% of the MAN-FAT subgroup were.

**Table 3:** Cross-tabulation of subgroup (i.e. ‘anxious-depression’ (ANX-DEP); ‘mania-fatigue’ (MAN-FAT); ‘developmental-psychotic’ (DEV-PSY)) by medication status

***Current medication***		***ANX-DEP (n* = *310)***	***MAN-FAT (n* = *152)***	***DEV-PSY (n* = *143)***	***Significance test***
NIL	Count	97	31	35	χ2 (2, 605) = 6.6 **[.037]**
	%	31.2%	20.3%	24.5%	
Any anti-depressant	Count	158	71	38	χ2 (2, 605) = 23.9 **[.000]**
	%	50.8%	46.7%	26.6%	
Any anti-psychotic	Count	68	74	87	χ2 (2, 605) = 73.5 **[.000]**
	%	21.9%	48.7%	60.8%	
Any mood stabilizer	Count	19	49	12	χ2 (2, 605) = 64.6 **[.000]**
	%	6.1	32.2	8.4	

The assessment of co-morbidities demonstrates that the DEV-PSY sub-group reported higher rates of ADHD (see Table 4), but there were no between group differences in reported rates of autistic spectrum disorders. Comorbid alcohol and other substance misuse rates were comparable across all groups (37-44%) as were the rates of nicotine, cannabis and alcohol use. The mean ages at first use (14–15 years) were similar across sub-types.

**Table 4:** Prevalence of comorbid diagnoses (top panel) and positive family history (bottom panel) of cases classified as: (i) ‘anxious-depression’ (ANX-DEP); (ii) ‘mania-fatigue’ (MAN-FAT); or (iii) ‘developmental-psychotic’ (DEV-PSY)

	***ANX-DEP***	***MAN-FAT***	***DEV-PSY***	***Significance test***
	***(n*** = ***310)***	***(n*** = ***152)***	***(n*** = ***143)***	***χ2 or ANOVA(2,df) [p]***
***Comorbidity***				
ADHD	13% (40/310)	11% (17/152)	24% (34/143)	12.4 [.015]
Autistic spectrum disorders	7% (20/310)	3% (5/152)	6% (8/143)	2.0 [.371]
***Substance use***				
Comorbid substance misuse	37% (115/310)	44% (67/152)	41% (59/143)	2.3 [.313]
Age first nicotine [% cases = users]	14.8 ± 2.5 [56%]	14.6 ± 3.1 [67%]	14.8 ± 2.8 [69%]	F (291) = 0.2 [.850]
Age first alcohol [% cases = users]	14.7 ± 2.4 [78%]	14.4 ± 2.2 [87%]	14.6 ± 3.3 [79%]	F (181.5) = 0.4 [.642]
Age first cannabis [% cases = users]	15.6 ± 2.3 [50%]	15.3 ± 2.7 [60%]	15.3 ± 2.4 [56%]	F (260) = 0.4 [.674]
***Family History***				
Depression (n/N)	56% (129/231)	54% (65/120)	24% (27/112)	33.1 **[.000]**
Anxiety (n/N)	22% (51/231)	34% (41/120)	14% (16/112)	13.2 **[.001]**
Bipolar (n/N)	12% (27/231)	28% (34/120)	7% (8/112)	24.3 **[.000]**
Schizophrenia (n/N)	7% (17/231)	18% (22/120)	23% (26/112)	18.2 **[.000]**
Substance misuse (n/N)	21% (49/231)	30% (36/120)	25% (28/112)	3.3 [.189]
Suicide (n/N)	6% (15/231)	4% (5/120)	6% (7/112)	0.8 [.672]

**Note**: ‘ADHD’ = Attention Deficit Hyperactivity Disorder.

For family history (see Table 4), the overall patterns of differences in rates of specific disorders suggested important distinctions between the sub-types. Specifically, the DEV-PSY group reported the lowest familial rates of depression (24%) and highest rates of schizophrenia/schizophreniform disorders (23%), while those in the MAN-FAT group reported the highest rates of anxiety (34%) and bipolar disorder (28%). The ANX-DEP group reported the highest rates of depression (56%). Family history of substance/alcohol misuse disorders and suicide were similar across subtypes.

As shown in Table 5, the multinomial logistic regression analysis demonstrated that the model that significantly differentiated between the groups (X^2^ = 133.89; df = 30; p < 0.001; Cox and Snell pseudo R^2^ = 0.39) included the following: age at presentation, HDRS, SOFAS, SIAS, family history of depression and bipolar disorder, and verbal learning, (with predicted IQ and family history of schizophrenia associated with p values of 0.054 and 0.058, respectively). The DEV-PSY subtype differed from both the MAN-FAT and the ANX-DEP groups on age at presentation to youth mental health services (DEV-PSY older), HDRS scores (less depressed) and SOFAS scores (more impaired). Furthermore, DEV-PSY cases were more likely than MAN-FAT cases to show impaired verbal learning (RAVLT sum score) and less likely to have a family history of bipolar disorders. Compared to the ANX-DEP, the DEV-PSY group were less anxious (SIAS), less likely to have a family history of depression, and more likely to have a lower predicted IQ level.

**Table 5:** Multinomial logistic regression analysis of characteristics that best discriminate cases classified as: (i) ‘anxious-depression’ (ANX-DEP) or (ii) ‘mania-fatigue’ (MAN-FAT) compared to ‘developmental-psychotic’ (DEV-PSY)

	**B**	**Sig.**	**Exp (B)**	**95% Confidence**	
				**Intervals for Exp (B)**	
**MAN-FAT**					
Age at presentation	-.22	.001	.80	.70	.92
SOFAS	.05	.04	1.05	1.01	1.09
HDRS	.12	.01	1.13	1.03	1.24
No family history of bipolar disorder^#^	−1.47	.03	.23	.06	.84
RAVLT sum score	.61	.04	1.85	1.04	3.29
**ANX-DEP**					
Age at presentation	-.16	.01	.85	.76	.95
SOFA	.052	.01	1.05	1.02	1.09
HDRS	.105	.02	1.11	1.02	1.21
SIAS	.038	.01	1.04	1.01	1.07
No family history of deppression^#^	−1.16	.01	.31	.13	.76
Predicted IQ	.046	.03	1.05	1.00	1.09

Note: Exp (B) > 1.0 indicates higher; Exp (B) < 1.0 indicates lower.

^#^ a negative value for B and an odds ratio (Exp (B)) <1.0 indicates lower proportions for the factor e.g.

‘No Family History’ indicates that the group is more likely to have a positive family history.

## Discussion

Before discussing the findings in detail, it is useful to highlight two issues. First, this cohort of young people has significant levels of symptoms, functional impairment, distress and suicidal ideation that warrant clinical intervention; and that this need for care and treatment is apparent whether or not the presenting symptoms reached current thresholds for diagnostic caseness [66-68]. Second, this study attempts to avoid one of the commonest problems encountered in assessing the clinical, familial and neuropsychological profile of young people with emerging mental disorders, namely the over-reliance on positive DSM or ICD-like ‘case’ versus normal ‘control’ strategies. Comparing a single diagnostic subgroup with a non-clinical, ‘healthy’ control group is of limited value when the ultimate goal is to identify the characteristics that best discriminate between clinical syndromes and/or will predict the future illness trajectories of young people who present for care. Also, a simple case–control approach will not help in attempts to identify biomarkers of transition from sub-threshold disorders to full-syndrome disorders, nor of future homotypic or heterotypic disease progression. As clinicians increasingly attempt the early identification and treatment of young people presenting in the initial stages of mental disorder (typically stage 1b or stage 2), it is likely that studies utilizing ‘positive case-positive control’ designs (i.e. case versus case) will become increasingly important [16].

In this study we examine the similarities and differences between three putative developmental trajectories that lead to adolescent-onset disorders – namely, ANX-DEP, MAN-FAT, and DEV-PSY disorders. These sub-types draw, in part, on historical distinctions between depressive disorders driven by heightened sensitivity to environmental stressors thresholds, circadian-based mood disorders (including bipolar spectrum and atypical depression syndromes) and psychotic disorders with or without affective components (broadly defined and often linked to evidence of earlier deviations in brain development). The study also seeks to apply these concepts to young people with attenuated syndromes before the symptoms progress to classical, more easily distinguished, or more stable syndromes. As such, this tripartite model builds on established diagnostic categories, extending them to sub-threshold presentations via the use of a clinical staging framework, and further refining them by classifying cases on the basis of a putative clinical phenotype linked to a proposed underlying pathophysiology and illness trajectory. However, it differs from other historical approaches to classifications that have traditionally given more weight to psychotic symptoms - because these were perceived as most relevant to diagnostic hierarchies [4,13,69]. Our approach is supported by contemporary evidence that increasingly emphasizes the importance of developmental psychopathology and highlights the key role of anxiety and depression in the evolution of most adult prototype severe mental disorders and offers a rationale for giving greater priority to these symptoms [22,33,70].

The differential demographic (older, more male population in the DEV-PSY subgroup), developmental (lower IQ and higher rate of diagnosed ADHD in the DEV-PSY subgroup), and phenotypic (more severe depression, suicidal ideation and anxious features in the ANX-DEP and MAN-FAT subgroups; more social avoidance in the ANX-DEP subgroup) characteristics are consistent with the findings of other longitudinal, clinical and epidemiological studies and lend support to the proposed model of sub-types [15,31-34,40,41,70,71]. Further, when we limited the analyses to cases with early or ‘attenuated’ or sub-threshold syndromes only, these differential patterns were for the most part preserved, suggesting that they potentially represent important discriminating features. Other characteristics, such as education, general psychiatric symptom levels, autistic spectrum or concurrent substance misuse disorders (which have been reported to be associated with onset of psychosis or severe mood disorders when examined in case–control studies), did not discriminate between the three sub-groups, again supporting the findings from studies that employ positive case-case designs. Whilst some between group differences may have been influenced by the reasons for presentation to youth mental health services (e.g. DEV-PSY may present because of concerns regarding impaired level of functioning; ANX-DEP may present because of levels of distress), the proposed sub-types were also differentiated on the basis of family history of mental disorders and neuropsychological testing. This is important as these are widely regarded as two of the most robust and frequently utilized cross-sectional strategies currently available to test the validity of proposed clinical sub-groups [34,43,63,71].

The patterns of neuropsychological impairment strongly differentiated the DEV-PSY group from the mood sub-types, particularly with regards to verbal learning and memory, and to a lesser extent to processing speed. These profiles are consistent with other neuropsychological studies in young people across the mood disorders and psychosis spectrum [34,63]. The differences may be partly explained by the fact that the DEV-PSY group had fewer cases classified as sub-syndromal (stage 1b) and a significantly lower mean IQ. However, the difference in verbal learning remained significant in the multinomial regression when other factors were taken into account and this abnormality has been reported as a key difference between individuals at high risk of psychosis who do not make the transition to illness compared to those who make the transition to psychosis (e.g. [72]). The findings are also consistent with structural brain imaging studies in young persons with similar phenotypic characteristics that suggest greater temporal lobe changes in those with psychotic features (as compared with frontal lobe features that appear to be common across the mood disorder and psychotic spectrum) [31,38,72].

Finally, it is important to acknowledge some important study limitations. First, the cohort represents a sub-sample of referrals to youth mental health services and biases in referral processes and/or the exclusion of cases that could not be classified could have influenced our findings. Although we have evidence that the sample reflects the case mix in the clinical service, we cannot rule out biases entirely, so replication of the study is necessary [66-68]. It is also worth noting, however, that this study was not designed to test the viability of such a differential developmental model in the general population. Second, the small proportion of DEV-PSY cases rated as stage 1b means some of the analyses may be less reliable for this sub-group. Clearly, studies with much larger sample sizes are needed to address this issue, particularly with respect to positive case-positive control designs where the it is important to have a good representative of both ‘sub-syndromal’ and ‘full-threshold’ subjects who may have differing interpretations of their own early stages of illness. Third, in order to maximize participation in the study, we limited the assessment of some aspects of the developmental and illness history, so again this may have implications for the delineation of clinical subtypes [18]. Fourth, we did not analyze treatment patterns (as we do not use this to define/confirm the groups), but these may of course have modified the current level of reported symptoms or impacted on neuropsychological tests. The extent to which medication use may also lead to misattribution of sub-type is difficult to determine empirically. However, it is possible that a small number of cases who would otherwise be allocated to the MAN-FAT or DEV-PSY group were assigned to the default ANX-DEP. It is notable that, as individuals were assessed at the time of presentation to the youth services, the majority of cases were not in receipt of long term and/or high dosages of antipsychotic, antidepressant or mood-stabilizing medications. Finally, this report is only the first step in the development of the tripartite sub-type model. The present study was reliant largely on cross-sectional comparisons of key phenotypic, clinical and neurocognitive characteristics. Furthermore, this study had relatively large subgroup sizes and therefore the functional and/or clinical relevance of key measures (e.g. significant predictors with narrow confidence intervals) need to be further evaluated. Future studies may benefit from the exploration of other factors, such as early life trauma, social support and/or metacognitive capacities to differentiate these subgroups. Such measures may help explain, for example, why the DEV-PSY group reported lower levels of depressive symptomatology compared to the other groups. It is possible this subgroup of subjects express their distress differently or have an altered capacity to articulate mood or other emotional symptoms. Clearly the next major step is to follow this cohort prospectively. This will allow us to examine the longitudinal characteristics of the model, to test its capacity to predict course of illness, and to examine any differential responses to personalized treatments selected on the basis of the proposed underlying pathophysiology and developmental trajectories.

## Conclusions

This cross-sectional study of 605 young people presenting for mental health care provides provisional support for a model that differentiates subjects into three subgroups (anxious-depression, mania-fatigue, developmental-psychotic) based largely on differential childhood and adolescent trajectories. While the model shares key concepts with more traditional contrasts between psychotic and non-psychotic disorders, it proposes the differentiation of two mood disorder types based largely on changes in motor or psychic activation or the presence of prior or concurrent anxiety. While this tripartite model is supported by key differences in clinical, family history and neuropsychological characteristics, relevant prospective and comparative intervention studies are now required to test its predictive validity.

## Abbreviations

ADHD: Attention deficit hyperactivity disorder; ANX-DEP: Anxiety-depression; ANOVA: Analysis of variance; AUDIT: Alcohol use disorders identification test; BPRS: Brief psychiatric rating scale; CBT: Cognitive behavioural therapy; DEV-PSY: Developmental-psychotic; DSM: Diagnostic and statistical manual of mental disorders; ECT: Electroconvulsive therapy; HDRS: Hamilton depression rating scale; ICD: International classification of diseases; IQ: Intelligence quotient; K-10: Kessler-10; MAN-FAT: Mania-fatigue; MRI: Magnetic resonance imaging; MNLR: Multi-nomial logistic regression; NIMH: National institute of mental health; RDoC: Research domain criteria; SIAS: Social interaction anxiety scale; SOFAS: Social and occupational functioning assessment scale; SPSS: Statistical product and service solutions; SSRI: Selective serotonin reuptake inhibitor; TMT: Trail-making test; RAVLT: Rey auditory verbal learning test; WRAT: Wide range achievement test; WTAR: Wechsler test of adult reading; YMRS: Young mania rating scale.

## Competing interests

Professor Hickie is a Board Member of Psychosis Australia Trust. From 2012, he has been a Commissioner in Australia’s new National Mental Health Commission. He was until January 2012 a director of headspace: the national youth mental health foundation. Prof Hickie was previously chief executive officer (till 2003) and clinical adviser (till 2006) of beyondblue, an Australian National Depression Initiative. He is supported principally for clinical research in depression and health services and population health initiatives related to anxiety and depression by an NHMRC Australian Medical Research Fellowship (2007–12) and now by an NHMRC Senior Principal Research Fellowship (2013-17). He has led projects for health professionals and the community supported by governmental, community agency and pharmaceutical industry partners (Wyeth, Eli Lily, Servier, Pfizer, AstraZeneca) for the identification and management of depression and anxiety. He has received honoraria for presentations of his own work at educational seminars supported by a number of non-government organisations and the pharmaceutical industry (including Pfizer, Servier and Astra Zeneca). He has served on advisory boards convened by the pharmaceutical industry in relation to specific antidepressants, including nefazodone, duloxetine, and desvenlafaxine. He leads an investigator-initiated study of the effects of agomelatine on circadian parameters (supported in part by Servier but also by other NHMRC funding) and has participated in a multicentre clinical trial of agomelatine effects on sleep architecture in depression and a Servier-supported study of major depression and sleep disturbance in primary care settings. In addition to national and international Government-based grant bodies, investigator-initiated mental health research at the BMRI he has been supported by various pharmaceutical manufacturers (including Servier and Pfizer) and not-for-profit entities (including the Heart Foundation, beyondblue and the BUPA Foundation).

Dr Daniel Hermens is currently supported by a grant from the NSW Ministry of Health, Mental Health and Drug & Alcohol Office as well as an NHMRC Australia Fellowship (awarded to Professor Hickie). He has received honoraria for educational seminars from Janssen-Cilag and Eli Lilly.

Professor Jan Scott is an unpaid advisor or committee member for the following: International Early Psychiatry Association, International Societies of Affective Disorders and of Bipolar Disorders, UK Bipolar Organization (Manic Depression Fellowship), International Bipolar Disorders Fellowship. She has ongoing funding from the UK MRC, UK NHS FSF and UK NHS RfPB programmes for research on mood disorders, bipolar II disorders, predicting transition in ‘at risk’ populations and development of psychological therapies. Her other mental health research has been supported by a UK NHS SDO grant and funds provided to the NTW NHS Trust by Astra-Zeneca and Janssen-Cilag for investigator initiated studies on mood disorders, psychosis and medication adherence. She has received honoraria for presentations of her own work at educational seminars supported by a number of non-government organizations and the pharmaceutical industry and has also served on advisory boards convened by the pharmaceutical industry mainly in respect of her research interest in medication adherence (including Astra-Zeneca, Janssen-Cilag, Lundbeck, Pfizer, Sanofi-Aventis and Servier).

Dr Elizabeth Scott is the (unpaid) Clinical Director of Headspace Services at the BMRI, the (unpaid) Co-ordinator of the Youth Mental Health Research Program at the BMRI, and Deputy Director of St Vincent’s Private Hospital Young Adult Mental Health Unit. She has received honoraria for educational seminars related to the clinical management of depressive disorders supported by Servier and Eli-Lilly pharmaceuticals. She has participated in a national advisory board for the antidepressant compound Pristiq, manufactured by Pfizer.

## Authors’ contributions

IBH and DFH prepared the initial draft manuscript. IBH and EMS supervised and verified all clinical assessments. DFH and JS conducted the statistical analyses. IBH, DFH, SN and EMS conceived the study design. JS, AG and NG provided key interpretation of the clinical data and participated in various aspects of the study design and analysis. All authors contributed significantly to the interpretation of the data as well as having read and approved the final manuscript.

## Pre-publication history

The pre-publication history for this paper can be accessed here:

http://www.biomedcentral.com/1471-244X/13/303/prepub
